# Effects of COVID-19 in Endocrine Patients: A Cross-Sectional Study

**DOI:** 10.3390/medicina58101375

**Published:** 2022-09-30

**Authors:** Elisabetta Morini, Giuseppa Maresca, Lilla Bonanno, Francesco Corallo, Viviana Lo Buono, Maria Cristina De Cola

**Affiliations:** IRCCS Centro Neurolesi Bonino-Pulejo, S.S. 113 Via Palermo, C.da Casazza, 98124 Messina, Italy

**Keywords:** COVID-19, SARS-CoV-2, daily habits, sleep disorders, anxiety, endocrine patients

## Abstract

**Introduction:** Home confinement due to COVID-19 lockdown led to changes in daily routines, including social interactions, as well as restrictions on the possibility of playing sports and eating habits. These changes could have a greater impact on patients suffering from chronic diseases, such as endocrine patients, especially in emotional and behavioral dimensions. **Materials and Methods:** This study aimed to assess the effects of COVID-19-induced quarantine on daily habits in a group of patients with endocrine disorders, focusing on food consumption, eating habits and sleep during the confinement. Eighty-five endocrine patients were enrolled. A structured interview was administered to investigate socio-demographic information, general medical conditions, and habits adopted during quarantine. All patients underwent the Spielberger State Anxiety Inventory (STAI-Y1) to assess state anxiety. **Result:** Results showed that subjects mainly had a sedentary lifestyle. We found a significant increase in the number of cigarettes in smokers and in meals consumed during confinement, as well as a high rate of sleep disturbance, especially insomnia. Notably, physical well-being resulted to be a predictive factor (OR = 0.38; 95%CI = [0.95,0.66]), whereas anxiety was a risk factor for sleep disorder (OR = 1.22; 95%CI = [1.10,1.40]), as was working in public and private offices and being a student. **Conclusions:** Changes in daily habits were likely due to the alterations in routine, resulting in greater boredom and inactivity during the day. In addition, future research should focus on the importance of patient adherence to therapy.

## 1. Introduction

In autumn 2019, China was affected by Severe Acute Respiratory Syndrome Coronavirus 2 (SARS-CoV-2). It reached Italy at the end of February 2020, hitting primarily Lombardia and then spreading throughout the territory [1]. As with other respiratory pathogens, the virus is transmitted from human to human, so isolation represented the best way to contain this epidemic. On 11 March 2020, the World Health Organization (WHO) declared the “pandemic state” leading governments of the mainly affected countries to impose strict confinement on their citizens. In Italy, on 8 March 2020, the Government had already established extraordinary measures to limit viral transmission. Thus, population quarantine was implemented from 9 March to 3 May 2020 [2]. Some new rules have been imposed, such as working from home and closing schools, shops, restaurants and any business or service considered non-essential in order to slow down the spread of the contagion and thereby prevent the collapse of health care systems [3]. This event exerted the strongest impact on life of every individual on many levels (personal, work, social, economic and psychological) since World War II. As a result, these measures had a great impact on population’s general health; therefore, it was necessary to create screening and treatment programs to ensure the population’s and workers’ health [4].

After this national lockdown, a 7-to 21-day quarantine became mandatory for all virus-positive subjects, until June 2022. All of these impositions have changed some daily routines, including social interactions, how to play sports, and eating habits. Although these effects are still visible more than two years later [5], the most devastating effects were seen in the first phase of the pandemic. The sudden interruption of work routine has caused boredom in many cases, and continued exposure to information about COVID-19 from the media has been stressful. Both these conditions lead people to eat too much, preferring sugar-rich “comfort foods”, but also to upset their circadian rhythms, which are no longer punctuated by daily activities [6]. A condition that the pandemic has exacerbated enough to cause a 36% increase in symptoms associated with eating disorders and a boom in hospitalizations, which have increased by 48%. A domino effect mainly has shifted in patients with bulimia, anorexia nervosa, and other food-related conditions [7]. Primary prevention interventions focused on social distancing, as well as hand, personal and environmental hygiene; little, however, has been disseminated on eating behaviors [8].

Based on the information derived from experiences with COVID-19 patients worldwide to date and from previous epidemic situations such as SARS, in obese and/or diabetic patients there is a tendency for a more likely evolution to more severe forms (pneumonia or respiratory failure) [9]. At the same time, studies showed that dysfunctional eating behaviors are often related to endocrine disorders [10,11]; endocrine signals emerged as a particularly important aspect of eating physiology [12]. Thus, the malfunction of the endocrine system can have important repercussions on nutrition, leading to the possibility of higher risk of contracting a severe form for this patient population although available data do not allow us to stratify within individuals with these diseases the categories most at risk. Certainly, the greatest risk is for male patients over 65 years of age with the presence of comorbidities, such as hypertension and cardiovascular disease, while there is equal evidence that severe forms of COVID-19 are rare in young and pediatric patients [13]. Another condition particularly at risk is immunosuppressed patients because they are on high-dose corticosteroid therapy or because they have a form of hypercortisolism of endogenous origin (Cushing’s syndrome). The situation of these patients is particularly critical because they also frequently have overweight/obesity, hypertension, and diabetes mellitus [14].

There is an extensive literature on the effects induced by dietary changes in endocrine patients, but little attention has been paid to nutrition education and the behavioral and emotional traits of the quarantined population [15]. This study aimed to observe how emotional and behavioral effects might have affected the habits of patients with endocrinological disorders during the COVID-19 pandemic.

## 2. Materials and Methods

In this cross-sectional study, outpatients of the endocrinology clinic of the IRCCS Centro Neurolesi “Bonino-Pulao” of Messina were contacted by phone from May to June 2020 to complete a structured interview on food-related habits adopted during the COVID-19 lockdown. They had been followed by the endocrinologist during the confinement by means of consultation calls occurred every 15/20 days.

**Figure 1:**
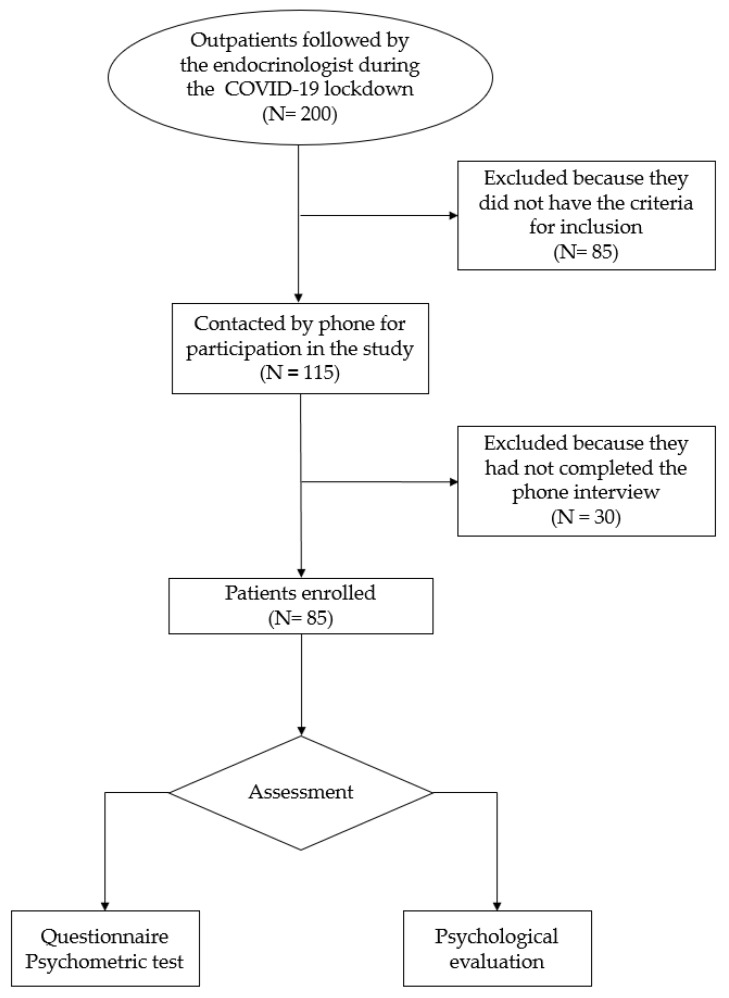
Flow-chart of the patients’ enrollment.

The patients were initially screened according to the following inclusion criteria: (i) patients with thyroid dysfunction, patients with metabolic osteopathy; (ii) age over 18 and under 80. Exclusion criteria were: (i) neurological or psychiatric disorders; (ii) story of eating disorders or malnutrition; (iii) first outpatient visit. All patients who spontaneously decided to fill out the questionnaire were enrolled (Figure 1). Thus, eighty-five subjects (18 males and 67 females) accepted to participate in this study. The structured interview included three sections: the first including socio-demographic information; the second relating to general medical conditions (as the presence of pathologies and comorbidities); the last section concerning habits adopted during the quarantine, especially dietary behavior, social interactions, sleep routine, physical activity, and the adoption of healthy habits (e.g., taking supplements such as psyllium, glucomannan, dietary fiber, sitosterol and chromium, eating healthier, reducing salt or soft drinks). Moreover, the Spielberger State Anxiety Inventory (STAI-Y1) was administered to assess anxiety caused by a specific condition (state anxiety) [16], which was our primary endpoint. We chose this psychometric test because among all the tests that measure anxiety, this one discriminates state anxiety that could be related to problems that are susceptible as a result of COVID-19. In addition, this instrument can measure state anxiety, which could help us to exclude patients with mental disorders.

The study protocol was approved by the Local Ethics Committee of IRCCS Centro Neurolesi Bonino-Pulejo of Messina (Protocol number 23/2020) and was conducted according to the 1964 Declaration of Helsinki and its later amendments. All subjects signed informed consent and their anonymity was guaranteed through a code matching the interview to the STAI-YI score.

### Statistical Analysis

To detect statistical significance, considering a significance level of 95 percent and a confidence interval of 10, a total of 65 subjects were needed from a population of 200 patients. Estimation of the sample size to be interviewed to obtain results that most accurately reflect the target population was done using Creative Research Systems survey software (https://www.surveysystem.com/sscalc.htm, accessed on 2 June 2012).

Descriptive analysis of cohort was reported on demographic and clinical variables. Continuous variables were expressed as median (first-third quartile), whereas categorical variables in frequencies and percentages. A nonparametric analysis was carried out because the results of the Shapiro normality test indicated that most of the target variables were not normally distributed. Thus, the Wilcoxon sum rank test was used to compare continuous variables, whereas the 𝜒^2^ test with continuity correction was used to assess for statistical differences in proportions. Correlations between variables were assessed by mean of the Spearman’s rank correlation coefficient. Since one of the predominant disorders detected in our sample was sleep, finally, a multiple logistic regression was performed with the aim of investigating possible predictors of sleep disorders among clinical (anxiety, comorbidity, physical wellness) and socio-demographic (age, gender, education, type of job) variables. We applied a backward elimination stepwise procedure for the choice of the best predictive variables according to the Akaike information criterion (AIC). Analyses were performed using an open source R3.0 software package (R Foundation for Statistical Computer, Vienna, Austria). Statistical significance was set at *p* < 0.05.

## 3. Results

### 3.1. General Clinical Picture

Participants had a median age of 61 (52–69) years and education of 13 (8–13) years. A more detailed description of their demographic characteristics is reported in Table 1. Moreover, around 94% of the subjects suffered from at least one chronic disease, indifferently by gender (𝜒^2^(1) < 0.001; *p* = 0.99). Nobody resulted to be infected by the COVID-19 virus, and only 6% had a family member or an acquaince infected.

### 3.2. Lifestyle and Habits

Subjects had mainly a sedentary lifestyle: 52.9% did not practice sport, 10.6% practiced sport less than one hour per week, 31.8% practiced sport more than 2–3 h per week, 4.70% practiced sport 4 h per week. Although 78.8% of subjects were no-smokers, about 72.2% of smokers had a significant increase in the daily number of cigarettes (𝜒^2^(1) = 51.69; *p* < 0.001). About 63.5% of subjects declared an increase of meals consumed during the COVID-19 quarantine, with an increase of 1–2 kg in 63.5% of subjects, and of more than 3 kg in 30.6%. Indeed, around 62.4% of subjects consumed several snacks and light meals, besides the three main meals (i.e., breakfast, lunch, dinner). However, only 33% of subjects declared of being motivated to follow a healthy diet after the quarantine.

### 3.3. Anxiety and Physical Wellness

The mean physical wellness of the interviewed, measured on a 0–10 range scale, was 6.3 ± 1.4, which resulted to be correlated with age (r = −0.24; *p* < 0.05) and education (r = 0.25; *p* < 0.05). No significant correlation between the physical wellness and anxiety emerged (r = −0.14; *p* = 0.2). However, around 34% of subjects presented anxiety, although this was only significantly associated with the presence of sleep disorders (𝜒^2^(3) = 18.86; *p* < 0.001). Notably, the proportion of insomnia in anxious subjects was significantly higher than in people without anxiety symptoms (𝜒^2^(1) = 6.8; *p* < 0.01).

### 3.4. Anxiety and Sleep Disorders

**Figure 2:**
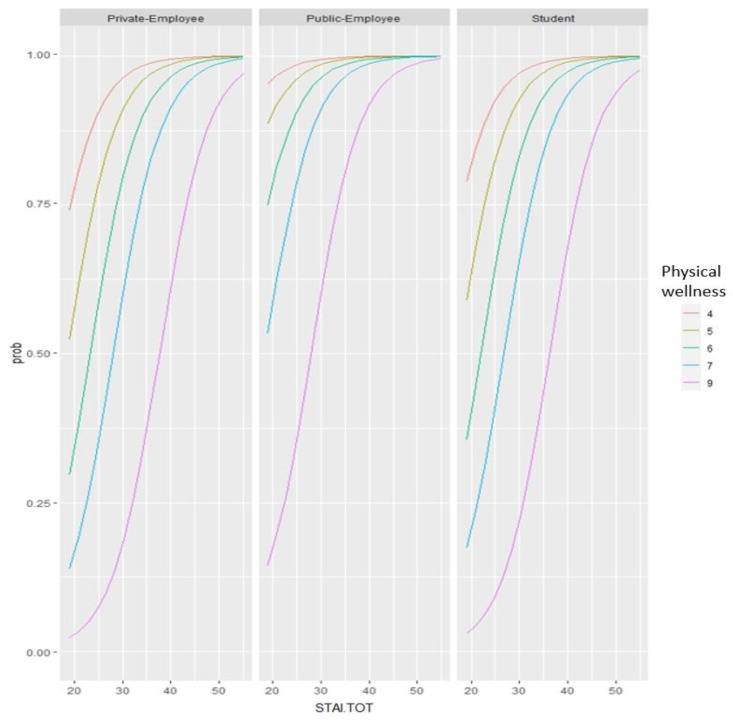
Graphic representation of the regression (only significant predictors) of sleep disorder in COVID-19 quarantine.

We found a high rate of sleep disorder occurrence (62.4%), especially insomnia (36.5%). Thus, we divided the cohort in two groups by the presence/absence of sleep disorder. The backward elimination stepwise procedure identified the logistic model including as predictors of physical wellness, anxiety, and type of job. Notably, physical wellness resulted to be a predictive factor (OR = 0.38; 95%CI = [0.95,0.66]), whereas anxiety (OR = 1.22; 95%CI = [1.10,1.40]) was a risk factor for sleep disorder, as well as working in public and private offices and being a student (Table 2, Figure 2).

Finally, in Table 3 there are summarized the major findings relating to sleep disorders and the other variable analyzed, also considering the whole cohort.

## 4. Discussion

The pandemic has placed the emphasis on issues related to coronavirus infection without considering the needs of the population in terms of physical and mental well-being. Much attention was paid to the frail category, thus the majority of the population in many cases found themselves uncovered from adequate medical assistance [17]. The aim of our study was to observe how the emotional and behavioral aspects elicited by COVID-19 affected their habits and behaviors of daily life. Considering that the endocrine patient population is particularly susceptible to dietary changes and stress, we wanted to observe it in more depth, to make considerations about the implications of health, understood in a more complex and articulated sense. Although many studies have focused on dietary changes or behavioral disorders in endocrine patients, the original aspect of our work was to create an ad hoc questionnaire on our patients from a knowledge base. In addition, a multidisciplinary team was responsible for the evaluation of the patient taken in charge.

The literature has reported an increase in psychiatric symptoms in the western population due to the radical and unexpected change of habits and freedom [2,6]. The main stressors were found to be the duration of the quarantine, fears of infection, frustration and boredom, inadequate supplies and insufficient information [17]. Italy was the first European country to be severely affected by COVID-19, with a higher distribution of cases in Lombardy, Emilia Romagna and Veneto regions, and was also the first European country to adopt a national lockdown during the pandemic, i.e., an emergency protocol requiring a nation’s citizens to stay at home during the outbreak of coronavirus. The study by Gualano et al. [18] showed that the general Italian population reported a high prevalence of mental health problems during the last weeks of closure. Maugeri et al. [19] found a lower percentage of cases in Sicily, with a mortality risk stably around 0.7%. One plausible explanation is that the Sicilian regional health care system has not experienced the same emergencies observed in northern Italian regions. For this reason, we could hypothesize that the emotional effect was smaller in Sicily than in other regions, and this is the reason our findings are in contrast with the rest of the literature [2,8,20]. Only part of our cohort experienced some significant changes in daily routine, and there were no significant results in the onset of psychiatric symptoms due to quarantine. On the contrary, sleep routine seem to have undergone a significant change. This fact can be explained either as a somatization of anxiety symptoms or as an effect of the changes imposed by isolation that may have altered the circadian rhythms. Indeed, insomnia could be a result of worries, tension, and alertness because of the adverse outcomes of this epidemic [21]. Anxiety was a predictor of insomnia symptoms, as well as working in public and private offices and being a student. Moreover, the change in sleep routines could also be due to alterations in daily habits, which led to increased boredom and inactivity during the day. These elements could be responsible for other significant data, such as an increase in meals eaten during the confinement (with a weight gain of Kg) and increased daily number of cigarettes in smokers. It would be interesting to investigate what factors resulted in a change in sleep routine: going to bed late, waking up late, sleeping in the afternoon, exercising, not feeling tired or being too bored, for example. We found no relationship between endocrine pathologies and changes in food consumption, daily habits or psychiatric symptoms. Therefore, we hypothesize that the presence of chronic diseases exerted no effects on the way the pandemic was coped with. Indeed, despite the drastic and sudden change, our patients did not experience trauma or changes in disease management, contrary to what other studies have claimed.

Probably, our contrasting results are due to the good management of their illness, also supported by the helpfulness of the health care team, who followed up with them by phone even during the lockdown. However, we did not study the patient–physician relationship and the role of individual members of the multidisciplinary team. The study also presents other limitations, including the lack of a questionnaire on adherence to pharmacological and non-pharmacological therapies, the small population size, and the lack of a control group. However, it was mainly due to the short time window used for the interviews, with the idea that immediately after lockdown the data would more closely reflect the behaviors of these patients. In addition, our intent was to help clinic patients and observe changes in eating and behavioral habits.

Future research should focus on the importance of adherence and patient care, as well as emphasizing prevention. The results of our study are very encouraging, because despite the pandemic, patients did not have a worsening of their clinical condition.

## 5. Conclusions

From our results, it is important to draw attention to nutrition and feeding disorders during the COVID-19 pandemic in endocrine patients for these main reasons:

- The risk of disease relapse or worsening;
- The increased risk of COVID-19 infection in individuals with eating disorders;
- The possible emergence of an ex-novo eating disorder or addictive behavior;
- The inadequacy of psychological and psychiatric treatments during the onset of COVID-19;
- Sleep disorders.

Although we are currently in a different phase of the COVID-19 pandemic, this study may focus attention on the future prevention of other adverse events and encourage the clinic to put the focus on the patient from a multidisciplinary perspective.

## Figures and Tables

**Table 1:** Demographic characteristics of the cohort. Significant differences are in bold.

Characteristics	All	Males	Females	*p*-Value
Participants, N (%)	85	18 (21.2)	67 (78.8)	-
Age (years)	61 (52–69)	68 (60–72)	60 (50–68)	0.02
Education (years)	13 (8–13)	8 (8–13)	13 (8–13)	0.39
Marital Status, N (%) Single	10 (11.8)	0	10 (14.92)	0.28
Married	69 (81.2)	18 (100)	52 (77.61)	
Separated/Divorced	3 (3.5)	0	2 (2.98)	
Widowed	3 (3.5)	0	3 (4.48)	
Children, N (%)				0.44
No	74 (87.1)	1 (5.55)	10 (14.92)	
Yes	11 (12.9)	17 (94.44)	57 (85.07)	
Family members, N (%)				0.71
1	6 (7.1)	1 (5.55)	5 (7.46)	
2	34 (40.0)	9 (50)	25 (37.31)	
>2	3 (52.9)	8 (44.44)	37 (55.22)	
Job, N (%)				<0.01
Pensioned	26 (30.6)	11(61.11)	15 (22.39)	
Unemployed	6 (7.1)	0	6 (8.95)	
Housewife	20 (23.5)	0	20 (29.85)	
Student	3 (3.5)	0	3 (4.48)	
Public Employee	13 (15.3)	2 (11.11)	11 (16.42)	
Private Employee	7 (8.2)	2 (11.11)	5 (7.46)	
Freelance Professional	2 (2.4)	1 (5.55)	1 (1.49)	
Artisan/Trader	5 (5.9)	2 (11.11)	3 (4.48)	
Laborer	3 (3.5)	0	3 (4.48)	

**Table 2:** Backward Logistic regression: significant predictors of sleep disorder in COVID-19 quarantine.

Predictors	Coefficient	Std. Err.	Wald z	Odds Ratio	[95% Conf. Interval]		*p*-Value
Physical wellness	−0.958	0.31	−3.12	0.383	0.95	0.66	<0.01
Anxiety	0.201	0.06	3.33	1.222	1.10	1.40	<0.001
Job—public employee	5.789	1.96	2.95	326.849	11.10	2674.69	<0.01
Job—private employee	3.832	1.86	2.06	46.136	1.65	291.58	0.04
Student	4.098	1.97	2.08	60.249	1.62	478.53	0.04

Pseudo-R2 = 0.57; Prob > χ^2^(10) < 0.001.

**Table 3:** Main cohort characteristics by sleep disorders. Significant differences are in bold.

Characteristics	All	Presence of Sleep Disorders	Absence of Sleep Disorders	*p*-Value
**Participants**	85	53 (62.4)	32 (37.6)	-
**Sex—Female**	67 (78.8)	44 (83.0)	23 (71.9)	0.34
**Comorbidity with other chronic diseases**	80 (94.1)	51 (96.2)	29 (90.6)	0.56
**Smokers**	18 (21.2)	8 (88.9)	5 (55.6)	0.29
**Sport before the pandemic**				0.50
<1 h/week	9 (10.6)	4 (7.5)	5 (15.6)	
>2–3 h/week	27 (31.8)	19 (35.8)	8 (25.0)	
>4 h/week	4 (4.7)	3 (5.7)	1 (3.1)	
**Nutrition during the lockdown**				0.57
More than before	54 (63.5)	35 (66.0)	19 (59.4)	
Less than before	6 (7.1)	4 (7.6)	2 (6.2)	
As before	24 (28.2)	14 (26.4)	10 (31.2)	
**Quality of nutrition during the lockdown**				0.05
Worse than before	52 (61.2)	36 (67.9)	16 (50.0)	
As before	27 (31.8)	12 (22.6)	15 (46.9)	
Better than before	6 (7.1)	5 (9.4)	1 (3.1)	
**Meals during the lockdown**				0.39
3 main meals	28 (32.9)	15 (28.3)	13 (40.6)	
3 main meals + 2 snacks	53 (62.5)	36 (67.9)	17 (53.1)	
2 main meals	4 (4.7)	2 (3.8)	2 (6.2)	
**Most consumed foods during the lockdown**				0.58
Fruits and vegetables	35 (41.2)	10 (18.9)	5 (15.6)	
Legumes and canned food	33 (38.8)	1 (1.9)	1 (3.1)	
Flour, frying, chips, rustics	15 (17.6)	19 (35.8)	16 (50.1)	
Sweets, chocolate, ice cream	2 (2.3)	23 (43.4)	10 (31.2)	
**Most consumed beverages during the lockdown**				**0.04**
Water	27 (31.8)	12 (22.6)	15 (46.9)	
Fruit juices	27 (31.8)	5 (9.4)	2 (6.2)	
Beer and wine/Hard liquor	24 (28.2)	14 (26.4)	10 (31.2)	
Carbonated soft drinks	7 (8.2)	22 (41.6)	5 (15.6)	
**Weight after the lockdown**				0.94
1–2 kg more than before	31 (36.5)	18 (33.9)	13 (40.6)	
1–2 kg less than before	13 (15.3)	8 (15.1)	5 (15.6)	
>2 kg than before	26 (30.6)	17 (32.1)	9 (28.1)	
As before	13 (15.3)	9 (17.0)	4 (4.0)	

Variables are expressed as frequencies (percentages).

## Data Availability

The datasets of the current study are available from the corresponding author upon reasonable request.
